# Relationship Between Generative AI Use and Life Satisfaction and the Mediating Role of AI Literacy Among Hong Kong Adults: Cross-Sectional Study

**DOI:** 10.2196/88362

**Published:** 2026-07-14

**Authors:** Ted CT Fong, Chee Hon Chan, Alex Pak Ki Kwok, Ryder TH Chan, Raymond Lap Ming Tang, Ming Wen, Paul SF Yip

**Affiliations:** 1 Social Science Research Centre Faculty of Social Sciences University of Hong Kong Hong Kong China (Hong Kong); 2 Centre on Behavioral Health Faculty of Social Sciences University of Hong Kong Hong Kong China (Hong Kong); 3 School of Governance and Policy Science Faculty of Social Sciences Chinese University of Hong Kong Hong Kong China (Hong Kong); 4 Department of Social Work and Social Administration Faculty of Social Sciences University of Hong Kong Hong Kong China (Hong Kong); 5 Hong Kong Jockey Club Centre for Suicide Research & Prevention Faculty of Social Sciences University of Hong Kong Hong Kong China (Hong Kong); 6 Department of Sociology Faculty of Social Sciences University of Hong Kong Hong Kong China (Hong Kong); 7 Research Hub of Population Studies Faculty of Social Sciences University of Hong Kong Hong Kong China (Hong Kong)

**Keywords:** age difference, AI literacy, digital divide, generative artificial intelligence, Hong Kong, older adults, structural equation model, life satisfaction, self-determination theory, technology acceptance model

## Abstract

**Background:**

Rapid technological advances have led to the development of generative artificial intelligence (GenAI). GenAI tools such as ChatGPT (OpenAI) and DALL-E (OpenAI) can generate text and images in response to prompts and have permeated various life domains. Existing literature has reported mixed relationships between GenAI use and life satisfaction.

**Objective:**

By synthesizing the Technology Acceptance Model and self-determination theory, this study aimed to examine the associations between GenAI use, perceived artificial intelligence (AI) usefulness, behavioral intention to use AI, AI literacy, and life satisfaction in general adults.

**Methods:**

A population-based survey recruited 1800 community-dwelling adults (mean age 49.3, SD 14.9 years, 995/1800, 55.3% women) via a 2-stage random sampling design in Hong Kong in spring 2024. Participants completed self-report measures on GenAI use and validated measures on perceived AI usefulness, behavioral intention to use AI, AI literacy, and life satisfaction. Multivariate analysis of covariance examined the gender and age differences in the study variables. Structural equation modeling was used to examine the associations between AI-related constructs, GenAI use, and life satisfaction in the whole sample and across gender and age subgroups.

**Results:**

Two-fifths (693/1800, 38.5%) of the sample reported GenAI use in the past year. There were significant age differences with declining trends in perceived AI usefulness, behavioral intention to use AI, GenAI use, and AI literacy from young adults to older adults. GenAI users with daily use of more than 2 hours reported significantly higher perceived AI usefulness, behavioral intention to use AI, AI literacy, and life satisfaction than nonusers. In the structural equation modeling, the direct effect of GenAI use on life satisfaction was not significant. There were significant positive indirect effects (αβ=0.186, 95% CI 0.134-0.242) from perceived AI usefulness to life satisfaction via behavioral intention to use AI, GenAI use, and AI literacy. Subgroup analyses found stronger total indirect effects in men (αβ=0.260, 95% CI 0.177-0.348) than in women (αβ=0.112, 95% CI 0.055-0.180) and in older adults (αβ=0.227, 95% CI 0.113-0.367) than in young adults (αβ=0.124, 95% CI 0.038-0.214).

**Conclusions:**

These findings synthesized the Technology Acceptance Model and self-determination theory and found a positive modest relationship between GenAI use and life satisfaction in Hong Kong adults. AI literacy showed a potential bridging role in the relationship between GenAI use and life satisfaction. Further longitudinal studies are needed to elucidate the causal direction and determinants of GenAI use and AI literacy.

## Introduction

Recent advances in artificial intelligence (AI) tools have transformed everyday life in sectors such as health care and public health [1]. In particular, generative AI (GenAI) tools such as ChatGPT (OpenAI) and DALL-E (OpenAI) can generate text, images, or other media in response to prompts and are increasingly used by the public [2] for purposes such as health information-seeking, self-care management, and counseling [3]. The usage rate, antecedents, and effects of GenAI use among the public warrant further investigation. The Technology Acceptance Model (TAM) offers a useful basis for predicting GenAI use. TAM has been widely applied in health informatics to explain users’ behavior toward technology and adoption of eHealth and digital interventions [4]. TAM posits that perceived usefulness and ease of use shape attitudes toward technology, driving behavioral intention and actual technology use [5]. Perceived AI intelligence, defined as the degree to which an AI interacts in a human-like manner and generates unscripted responses, has been found to predict GenAI use among higher education students [6]. However, a national cross-sectional survey of Chinese medical staff found disparities between high willingness to adopt AI and low actual use [7]. Qualitative studies among older adults revealed mixed perceptions regarding AI’s potential to enhance the quality of care and deep skepticism regarding usability, patient data privacy, and health misinformation [8,9]. The applicability of TAM in the GenAI context outside education settings warrants further study.

Based on self-determination theory, intrinsic motivation and psychosocial health could be improved by fulfilling 3 basic psychological needs (autonomy, competence, and relatedness) [10]. In the context of the use of AI tools, autonomy and competence could be enhanced by AI literacy, which denotes the knowledge and self-efficacy required to effectively understand, apply, and evaluate AI solutions as a socially responsible and ethical user [11]. With higher AI literacy, users can proactively seek the information they need, improving their sense of self-direction and making more informed choices in the workplace and personal life. In the digital era, AI literacy is considered essential for individuals’ self-management [11]. Given the conversational user interfaces that facilitate natural interaction between users and complex AI models [12], general users tend to find it easy to use GenAI and develop related literacy informally through repeated interaction with GenAI, learning from its outputs and refining prompts iteratively [13].

Furthermore, self-determination theory suggests that learning is more effective when driven by autonomous motivation rather than external pressure, which in turn could lead to better outcomes in GenAI use. A study among Saudi Arabian students linked ChatGPT use to higher self-esteem and academic engagement, finding that a sense of control over the tool positively predicted life satisfaction [14]. However, a recent survey in Slovenia [15] found a high usage rate of GenAI tools, yet GenAI use showed a significant but small correlation with life satisfaction, with one-fifth of the respondents reporting increased life satisfaction due to GenAI. Another study linked unfavorable perceptions of AI with lower life satisfaction across 39 European countries, independent of sociodemographic factors [16]. Limited research in the literature so far suggests mixed or small relationships between GenAI use and life satisfaction. On the one hand, GenAI can help automate routine tasks and reduce cognitive load, which in turn improves individual efficiency and autonomy. On the other hand, rapid integration of GenAI could lead to feelings of technology anxiety and insecurity and a loss of personal agency [17]. These competing mechanisms imply a potential dual role of GenAI and underscore the need for additional research.

The current literature on AI literacy is subject to methodological limitations that hinder its application to digital health policy. Prior works are based on convenience samples of specific subgroups, such as university students [18,19] or health care professionals [20,21], and focus on GenAI adoption in the medical field [22]. The narrow age ranges of existing samples prevent an in-depth examination of GenAI adoption as a social determinant of health across age cohorts, which limits the generalizability of the findings to the general population. Currently, there is a lack of representative population-based studies that investigate perceptions of AI, AI literacy, and GenAI use and their associations with outcomes across the human lifespan. Understanding how community-dwelling adults perceive and interact with GenAI is crucial for designing inclusive digital health interventions.

Besides, the digital divide in AI tool use is evident across demographic subgroups. Age showed significant negative correlations (*r*=–0.15 to –0.19) with AI literacy [21]. In Hong Kong, Shum and Lau [23] identified 3 latent profiles regarding attitudes toward AI among 740 middle-aged and older adults, with two-thirds (513/740, 69.3%) exhibiting ambivalent attitudes. Meanwhile, although a meta-analytic review of 43 studies from 31 regions concluded that women demonstrated higher objective digital literacy [24], they often reported lower AI literacy than men, with reasons including the underrepresentation of women workers in developing women-friendly AI and internalized societal gender bias [25]. A meta-analysis of more than 700,000 users found smaller associations for the predictors of the use of technology among women and older adults [26]. More evidence on age- and gender-related digital divide could shed insights into future policies that address the concerns of each subgroup and in turn reduce the associated digital health disparities.

Given these research gaps, this study aims to empirically test a model based on the TAM and self-determination theory in a population-based sample of community-dwelling adults in Hong Kong. First, we assessed population-level digital readiness in terms of 4 GenAI-related constructs, namely, perceived AI usefulness, behavioral intention to use AI, GenAI use, and AI literacy. Second, we examined the associations between perceived AI usefulness, behavioral intention to use AI, GenAI use, AI literacy, and life satisfaction, which could be a core and simple indicator of population psychosocial health [27] and health behaviors [28]. Third, we evaluated the potential bridging role of AI literacy in the pathway connecting GenAI use and life satisfaction by examining the associated indirect effects. Fourth, we explored the potential heterogeneity in the relationships between GenAI use, AI-related constructs, and life satisfaction across gender and age subgroups.

For the study hypotheses, we first hypothesized that men would score significantly higher in AI-related constructs, namely, perceived AI usefulness, behavioral intention to use AI, GenAI use, and AI literacy than women. Second, given the generational differences, we expected higher levels of GenAI use and AI-related constructs in young adults than in older adults. Third, guided by TAM, perceived AI usefulness and behavioral intention would show positive associations with GenAI use. Fourth, guided by self-determination theory, GenAI use would show positive associations with AI literacy and might show a small positive relationship with life satisfaction. Fifth, in synthesizing TAM and self-determination theory, AI literacy would act as a pathway connecting GenAI use and life satisfaction. This study extends prior work beyond convenience samples to a life course perspective, providing insights into public health informatics and a better understanding of the digital GenAI landscape in the community.

## Methods

### Study Design and Procedures

This study used a population-based, cross-sectional design in Hong Kong with a 2-stage, stratified random sampling design. In stage 1, we conducted random sampling at the living quarters level to select residential addresses built for habitation of one or more households, stratified by the 18 districts (geographic regions) and housing type. In stage 2, we randomly selected one eligible member per sampled household at the individual level using the “last birthday” method. Inclusion criteria were aged 15 years or older, residence in Hong Kong, and the ability to speak Chinese. Exclusion criteria included inmates, those living on vessels, and foreign domestic helpers. Invitation letters, which stated the study purpose, were sent to invite the household to participate in the survey. In case of nonresponses, on-site visits were conducted to recruit eligible members for face-to-face interviews. Voluntary informed consent was obtained. Data were collected online, by phone, or face-to-face in about 10 minutes, and the online system and interviewer ensured complete responses. A pilot study was conducted among 10 community adults in January 2024 and feedback from the respondents was used to refine the questionnaire and research process. Quality control included a random check of 208 interviews, with 90.8% passing the back-check criteria (at least 3 out of 4 questions in the quality back-check questionnaire were asked during the interview).

### Ethical Considerations

The study was reviewed and approved by the Human Research Ethics Committee of the University of Hong Kong (reference number EA200335). All participants were informed of the study objectives and provided informed consent before completing the survey. To minimize item-level missing data, the survey was programmed such that all questions required a response for the completion of the survey. Participation was voluntary, and participants could withdraw from the survey at any time. The final analytic sample contained no missing data since no respondents withdrew from the survey because of refusal to answer a question. Data were anonymized and all procedures contributing to this work comply with the ethical standards of the relevant national and institutional committees on human experimentation and with the Declaration of Helsinki in 1975, as revised in 2008. The study findings were presented in accordance with the STROBE (Strengthening the Reporting of Observational Studies in Epidemiology) reporting guidelines.

### Participants

Household data collection occurred between February and April 2024. We invited 3275 households, and 1800 eligible participants completed the survey. Reasons for nonresponse included noncontact (n=1399), refusal (n=63), and unoccupied households (n=13). According to the American Association for Public Opinion Research Response Rate 1 standard, the response rate was calculated by the formula: response rate = completed cases/(completed cases + partial cases + refusals + noncontacts + unknown eligibility cases) = 55.2%. The present sample was distributed across 18 districts (cluster size = 57-148) and 4 housing types: private ownership (43.9%), public rental (27.2%), subsidized ownership (19.3%), and private rental (9.6%). For the data collection mode, the number of responses collected through self-report questionnaire, phone interviews, and site visits were 553/1800 (30.7%), 765/1800 (42.5%), and 482/1800 (26.8%), respectively. The mean age was 49.3 (SD 14.9; range 17-95) years; 55.3% (995/1800) were women, 47.5% (855/1800) lived in urban districts, 68.6% (1235/1800) were married, 64.8% (1166/1800) were employed, and 85.9% (1547/1800) were born in Hong Kong. In the 2021 population census, Hong Kong residents had a median age of 46.3 (IQR 28-62) years and the labor force participation rate was 58%. The present sample showed similar sociodemographic profiles to the general Hong Kong population. Participants were categorized into 3 age groups: young adults (aged 17-39 years; n=521), middle-aged adults (aged 40-64 years; n=844), and older adults (aged 65-95 years; n=435).

### Measures

GenAI use was measured by the amount of time that the respondents spent on average per day using GenAI in the past year. In this study, GenAI was defined as deep learning models such as ChatGPT, DALL-E, and Claude that can generate text, images, or other media in response to prompts and excluded traditional virtual assistants such as Siri and Alexa. The time of GenAI use was answered on a 4-point scale: 0=“not used,” 1=“<1 hour/day,” 2=“1-2 hours/day,” and 3=“>2 hours/day.” A recent meta-analysis found moderate correlations between self-reported media use time and logged data and no statistically significant differences between single-item estimates and scale-based measures, or between measures of usage duration and usage volume [29]. This provides support for using self-reported daily time use. While the measure of AI usage was specific to GenAI, the following measures refer to AI in general.

AI literacy was evaluated using a 3-item short version of the 12-item Artificial Intelligence Literacy Scale (AILS) [30]. The AILS was developed in the Chinese context to measure users’ competence to understand, apply, and evaluate AI technology. This study selected 3 items from the AILS across 3 subscales: “I can distinguish between smart devices and non-smart devices” (Awareness), “I can skillfully use AI applications or products to help me with my daily work” (Usage), and “I can choose a proper solution from various solutions provided by a smart agent” (Evaluation). The use of a short version was to reduce participant burden and ensure brevity of the phone survey. The 3 items were answered on a 7-point Likert scale (1=“strongly disagree,” 2=“disagree,” 3=“slightly disagree,” 4=“neither agree nor disagree,” 5=“slightly agree,” 6=“agree,” and 7=“strongly agree”), with higher scores denoting greater AI literacy. Excellent reliability was found for the 3-item brief version of AILS (Cronbach α=0.93) in the present sample.

Perceived AI usefulness was evaluated by the following 2 items: “I believe that AI will improve my life” and “I believe that AI will improve my work”. Behavioral intention to use AI was assessed by the item: “I think I will use AI technology in the future.” These 3 items are selected from the validated 4-item Artificial Intelligence Attitude Scale (AIAS-4) [31] according to their theoretical alignment with the TAM. The AIAS-4 is a brief common measure of AI attitudes and has recently been validated in the Chinese context [32]. The items were rated on a 10-point Likert scale (1=“strongly disagree,” 2-3=“disagree,” 4=“slightly disagree,” 5-6=“neither agree nor disagree,” 7=“slightly agree,” 8-9=“agree,” to 10=“strongly agree”). Excellent composite reliability was found for perceived AI usefulness (Cronbach α=0.91) in the present sample.

Life satisfaction was measured by the 5-item Chinese version of the Satisfaction with Life Scale (SWLS) [33]. Example items include “I am satisfied with my life” and “So far I have gotten the important things I want in life.” The 5 items are rated on a 7-point Likert scale (1=“strongly disagree,” 2=“disagree,” 3=“slightly disagree,” 4=“neither agree nor disagree,” 5=“slightly agree,” 6=“agree,” and 7=“strongly agree”). The total life satisfaction score had a theoretical range from 5 to 35, with higher scores denoting greater life satisfaction. The SWLS showed excellent reliability (ω=0.93) in the present sample.

Sociodemographic characteristics included age, gender (men and women), education level (9-point scale; 1=“no formal education,” 2=“primary school,” 3=“lower secondary school,” 4=“upper secondary school,” 5=“diploma,” 6=“associate degree,” 7=“Bachelor degree,” 8=“Master’s degree,” and 9=“Doctoral degree”), place of birth (Hong Kong or other place), presence of chronic health conditions (such as restriction in body movement, mental disorder, hypertension, and diabetes mellitus), household size, work status (have full-time or part-time jobs), and monthly household income (12-point scale; 1=“less than HKD10,000,” 2=“HKD10,000-19,999,” 3=“HKD20,000-29,999,” 4=“HKD30,000-39,999,” 5=“HKD40,000-49,999,” 6=“HKD50,000-59,999,” 7=“HKD60,000-69,999,” 8=“HKD70,000-79,999,” 9=“HKD80,000-89,999,” 10=“HKD90,000-99,999,” 11=“HKD100,000-119,999,” and 12=“HKD120,000 or more”).

### Data Analyses

First, we examined the factorial validity of the 3 latent factors (perceived AI usefulness, AI literacy, and SWLS) using confirmatory factor analysis (CFA). Model fit was evaluated following standard criteria on fit indices [34]: comparative fit index (CFI) and Tucker-Lewis index (TLI) ≥ 0.95, and root-mean-square error of approximation (RMSEA) and standardized root-mean-square residuals (SRMRs) ≤ 0.06. Scalar measurement invariance of the factors was compared across gender and age groups between the configural invariance model (with free factor loadings and item intercepts) and the scalar invariance model (with equal factor loadings and item intercepts). Model comparison was performed using chi-square difference tests, Bayesian information criterion (BIC), and changes in fit indices with ΔCFI ≥ –0.01, ΔRMSEA ≤ 0.01, and ΔBIC ≥ 10, favoring the more parsimonious model [35].

Second, we conducted chi-square tests and multivariate analysis of variance (MANOVA) to compare sociodemographic profiles and GenAI use across gender and age groups. We performed MANCOVA to compare perceived AI usefulness, behavioral intention, AI literacy, and life satisfaction across GenAI use. MANCOVA controlled for the following sociodemographic covariates: gender, age, education level, place of birth, chronic condition, household size, work status, and household income. Effect sizes were denoted by partial eta-squares (η^2^) with 0.01, 0.06, and 0.14 denoting small, moderate, and large sizes, respectively [36]. Post hoc tests were conducted with Sidak adjustment. Statistical significance was taken at the .05 level.

**Figure 1:**
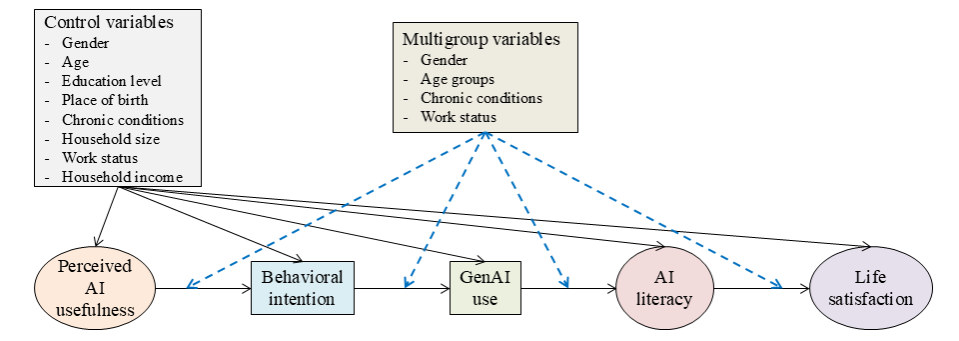
Conceptual model of this study on perceived artificial intelligence usefulness, behavioral intention to use artificial intelligence, generative artificial intelligence use, artificial intelligence literacy, and life satisfaction. AI: artificial intelligence; GenAI: generative artificial intelligence.

Third, we investigated the pathways specified in the conceptual model via structural equation modeling (SEM). Figure 1 illustrates the conceptual model of this study. Black arrows denote the paths tested in the main model. Dashed blue arrows refer to the moderating effects in multigroup analysis. Age, education level, place of birth, household size, and household income are the control variables in the SEM. Gender, chronic conditions, and work status act as the control variables in the SEM or the multigroup variables in the multigroup SEM.

In the main SEM, perceived AI usefulness, AI literacy, and life satisfaction were modeled as latent factors measured by the respective indicators. Behavioral intention to use AI and GenAI use were treated as an observed continuous and an observed ordinal variable, respectively. The model included the sociodemographic variables as covariates. We evaluated the indirect effect of perceived AI usefulness on life satisfaction through sequential mediators (behavioral intention, GenAI use, and AI literacy) using 10,000 bootstrap samples to account for the potential skewed distribution of the indirect effects. Standardized path coefficients and *R*^2^ denoted the effect sizes of the associations. Fourth, we conducted multigroup SEM across sociodemographic subgroups, namely, gender, age groups, chronic condition, and work status. In the multigroup SEM, we empirically tested the differences in the path coefficients using Model Constraint and compared the 95% CI of the indirect effects across subgroups.

Sampling weights were applied in the main analyses, namely, CFA, MANCOVA, and SEM, to align the sample distribution with the 2021 Hong Kong census distribution across 16 age × gender strata. These weights were not design-based sampling weights based on individual selection probabilities. CFA was conducted in Mplus 8.6 under Type = Complex using the robust maximum likelihood estimator [37]; MANCOVA was performed using the Complex Samples module in SPSS 28.0 (IBM Corp); and SEM was estimated under Type = Complex using the robust weighted least squares estimator to account for GenAI use as a categorical endogenous variable. To account for the complex survey design and nonindependence of observations, the residential district was entered as the cluster variable. Robust SEs were computed in CFA and SEM via the sandwich estimator, which was equivalent to Taylor series linearization.

## Results

### Factorial Validity and Measurement Invariance

The 3-factor CFA model showed a good fit to the data (CFI=0.985, TLI=0.978, RMSEA=0.044, and SRMR=0.027), with strong item loadings on perceived AI usefulness, AI literacy, and life satisfaction (λ=0.77-0.95, *P*<.001). Across gender, the scalar invariance model showed a comparable fit (CFI=0.985, TLI=0.982, RMSEA=0.041, SRMR=0.033, and BIC=51926) as the configural invariance model (CFI=0.986, TLI=0.980, RMSEA=0.044, SRMR=0.029, and BIC=52012) and the chi-square difference test was not significant (Δχ^2^_14_=14.5, *P*=.41). Across age groups, the scalar invariance model showed a comparable fit (CFI=0.977, TLI=0.975, RMSEA=0.047, SRMR=0.043, and BIC=51364) as the configural invariance model (CFI=0.981, TLI=0.974, RMSEA=0.049, SRMR=0.030, and BIC=51501). Despite a significant chi-square difference test (Δχ^2^_28_=54.4, *P*=.002), the substantially lower BIC for scalar invariance models supported equal loadings and intercepts across groups.

### Gender and Age Differences in Sample Profile

Tables 1 and 2 show the sample profile and comparison across gender. Overall, 61.5% (1107/1800) of the respondents did not use GenAI in the past year and 7.2% (130/1800) of them used GenAI for more than 2 hours per day. Men were significantly more likely to be working (*P*<.001) and had higher education level than women (*η^2^*=0.007, *P*<.001), and data collection was significantly more likely to be conducted via phone interviews (*P*=.002) in women than in men. No significant gender differences were found in place of birth (*P*=.91), chronic condition (*P*=.07), GenAI use (*P*=.11), and age (*P*=.56). Men showed significantly higher AI literacy (*η^2^*=0.008, *P*<.001) than women.

**Table 1:** Chi-square test on sample comparison across gender.

Variables			Overall (N=1800)		Men (n=805)		Women (n=995)		χ^2^ (*df*)			*P* value	
Born in Hong Kong, n (%)			1547 (85.4)		691 (85.1)		856 (85.6)		0.014 (1)			.91	
Have chronic condition, n (%)			387 (25.4)		189 (25.3)		198 (25.6)		3.38 (1)			.07	
Work status, n (%)													
	Yes	1167 (59.0)		593 (66.8)		574 (52.1)		49.8 (1)			<.001		
Data collection mode, n (%)										12.7 (2)			.002
	Self-administration	553 (31.1)		267 (33.0)		286 (29.4)							
	Site visits (face-to-face interviews)	482 (26.8)		233 (28.9)		249 (25.0)							
	Phone interviews	765 (42.1)		305 (38.1)		460 (45.6)							
GenAI^a^ use in the past year, n (%)										6.09 (3)			.11
	Never	1107 (61.9)		473 (57.9)		634 (65.6)							
	≤1 hour per day	423 (22.6)		196 (25.0)		227 (20.4)							
	1-2 hours per day	140 (8.1)		68 (8.2)		72 (8.0)							
	>2 hours per day	130 (7.4)		68 (8.9)		62 (6.0)							

^a^GenAI: generative artificial intelligence.

**Table 2:** Multivariate analysis of variance on sample comparison across gender^a^.

Variables	Overall (N=1800)	Men (n=805)	Women (n=995)	*F* test (*df*)	*η^2^*	*P* value
Age (years), mean (SD)	49.3 (14.9)	50.0 (14.9)	48.8 (14.8)	0.35 (1)	0.000	.56
Education level (1-9), mean (SD)	4.76 (1.76)	4.89 (1.81)	4.65 (1.71)	12.3 (1)	0.007	<.001
Household size (1-10), mean (SD)	2.95 (1.10)	2.85 (1.05)	3.03 (1.14)	4.25 (1)	0.002	.04
Household income (1-12), mean (SD)	4.80 (2.34)	4.91 (2.44)	4.71 (2.26)	6.03 (1)	0.003	.01
Perceived AI^b^ usefulness (0-10), mean (SD)	5.51 (2.11)	5.54 (2.20)	5.49 (2.04)	1.40 (1)	0.001	.24
Behavioral intention to use AI (0-10), mean (SD)	5.64 (2.32)	5.68 (2.41)	5.61 (2.24)	2.43 (1)	0.001	.12
AI literacy (1-7), mean (SD)	3.51 (1.50)	3.62 (1.54)	3.43 (1.46)	14.0 (1)	0.008	<.001
SWLS^c^ (5-35), mean (SD)	21.1 (5.69)	21.3 (5.62)	21.0 (5.75)	0.34 (1)	0.000	.56

^a^Unweighted frequencies and weighted percentages are presented, and the F test in multivariate analysis of variance adjusts for sampling weights.

^b^AI: artificial intelligence.

^c^SWLS: Satisfaction with Life Scale.

Tables 3 and 4 show the comparison among the 3 age groups. Older adults were significantly less likely to be working and to use GenAI in the past year (*P*<.001). There were moderate to strong age differences in education level, household income, perceived AI usefulness, behavioral intention to use AI, and AI literacy (η^2^=0.11-0.35, *P*<.001), with all variables declining with age. Life satisfaction did not differ across age groups. Supplementary analysis found no significant differences among the 3 data collection modes in other sociodemographic characteristics (η^2^=0.000-0.002, *P*=.17-.76), perceived AI usefulness (η^2^=0.001, *P*=.55), behavioral intention to use AI (η^2^=0.001, *P*=.62), GenAI use (χ^2^_6_=3.37; *P*=.76), AI literacy (η^2^=0.000, *P*=.79), and life satisfaction (η^2^=0.001, *P*=.46).

**Table 3:** Chi-square test on sample comparison across age groups.

Variables		Young adults (n=521)	Middle-aged (n=844)	Older adults (n=435)	χ^2^ (*df*)		*P* value	
Women, n (%)		302 (58.0)	458 (54.3)	235 (54.0)	2.15 (2)		.34	
Born in Hong Kong, n (%)		478 (91.7)	761 (90.2)	308 (70.8)	109.5 (2)		<.001	
Have chronic condition, n (%)		13 (2.5)	107 (12.7)	267 (61.4)	560.3 (2)		<.001	
Work status, n (%)								
	Yes	426 (81.8)	673 (79.7)	68 (15.6)	609.6 (2)		<.001	
Data collection mode, n (%)						5.66 (4)		.23
	Self-administration	178 (34.2)	244 (28.9)	131 (30.1)				
	Site visits (face-to-face interviews)	131 (25.1)	225 (26.7)	126 (29.0)				
	Phone interviews	212 (40.7)	375 (44.4)	178 (40.9)				
GenAI^a^ use in the past year, n (%)						168.1 (6)		<.001
	Never	242 (46.4)	493 (58.4)	372 (85.5)				
	≤1 hour per day	159 (30.5)	214 (25.4)	50 (11.5)				
	1-2 hours per day	63 (12.1)	65 (7.7)	12 (2.8)				
	>2 hours per day	57 (10.9)	72 (8.5)	1 (0.2)				

^a^GenAI: generative artificial intelligence.

**Table 4:** Multivariate analysis of variance on sample comparison across age groups^a^.

Variables	Young adults (n=521)	Middle-aged (n=844)	Older adults (n=435)	*F* test (*df*)	*η^2^*	*P* value
Age (years), mean (SD)	31.8 (5.83) ^d^	50.1 (7.08) ^e^	69.0 (4.89) ^f^	5054 (2)	0.85	<.001
Education level (1-9), mean (SD)	5.85 (1.40) ^f^	4.84 (1.60) ^e^	3.30 (1.39) ^d^	476.0 (2)	0.35	<.001
Household size (1-10), mean (SD)	3.05 (1.03) ^e^	3.00 (1.09) ^e^	2.74 (1.17) ^d^	29.1 (2)	0.03	<.001
Household income (1-12), mean (SD)	5.31 (2.27) ^e^	5.06 (2.32) ^e^	3.67 (2.10) ^d^	109.3 (2)	0.11	<.001
Perceived AI^b^ usefulness (0-10), mean (SD)	6.40 (1.83) ^f^	5.65 (1.95) ^e^	4.19 (2.09) ^d^	249.3 (2)	0.22	<.001
Behavioral intention to use AI (0-10), mean (SD)	6.53 (2.00) ^f^	5.86 (2.15) ^e^	4.15 (2.27) ^d^	244.6 (2)	0.21	<.001
AI literacy (1-7), mean (SD)	4.31 (1.26) ^f^	3.62 (1.34) ^e^	2.35 (1.33) ^d^	415.9 (2)	0.32	<.001
SWLS^c^ (5-35), mean (SD)	21.1 (5.86)	21.2 (5.75)	20.9 (5.38)	0.78 (2)	0.001	.44

^a^Unweighted descriptive statistics are presented, and the F test in MANOVA adjusts for sampling weights. Superscript letters denote significant post hoc differences among the age groups with d < e < f.

^b^AI: artificial intelligence.

^c^SWLS: Satisfaction with Life Scale.

### Comparison Across GenAI Use Levels

**Figure 2:**
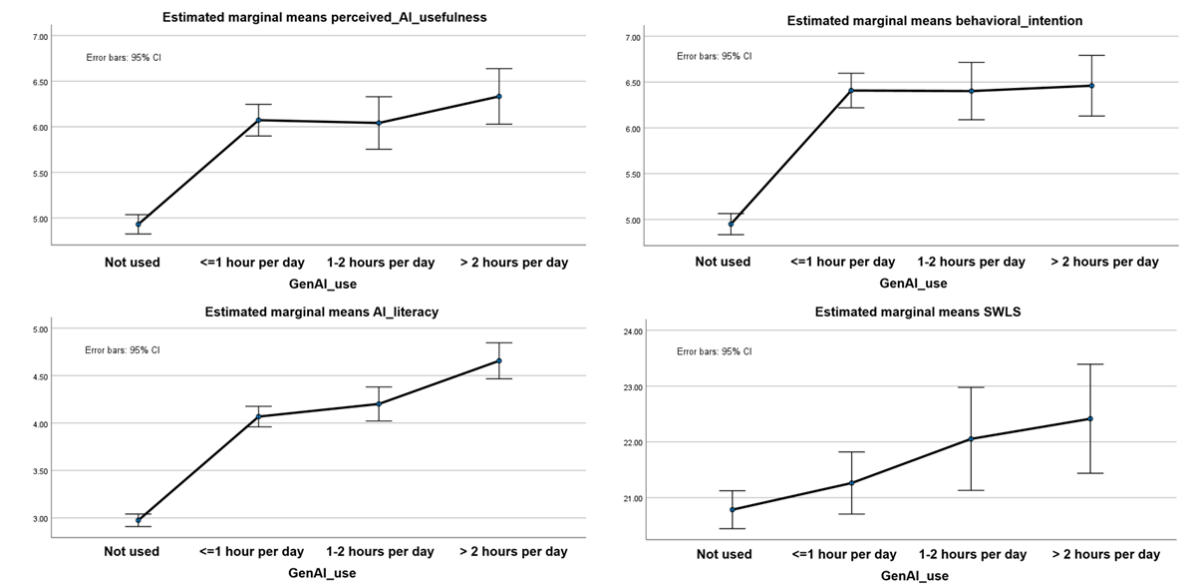
Plots of estimated marginal means and 95% CIs (in error bars) for perceived artificial intelligence usefulness, behavioral intention to use artificial intelligence, artificial intelligence literacy, and life satisfaction (Satisfaction With Life Scale) across generative artificial intelligence use levels. AI: artificial intelligence; GenAI: generative artificial intelligence; SWLS: Satisfaction with Life Scale.

MANCOVA across GenAI use revealed moderate between-subject difference for perceived AI usefulness and behavioral intention to use AI (η^2^=0.084-0.105, *P*<.001), a large difference for AI literacy (η^2^=0.209, *P*<.001), and a small difference for life satisfaction (η^2^=0.007, *P*=.005). Figure 2 displays the estimated marginal means of AI-related constructs and life satisfaction across the 4 levels of GenAI use. In the post hoc tests, GenAI nonusers showed significantly lower levels of perceived AI usefulness (mean difference –1.11 to –1.40, *P*<.001), behavioral intention to use AI (mean difference –1.45 to –1.50, *P*<.001), and AI literacy (mean difference –1.09 to –1.68, *P*<.001) than GenAI users, regardless of duration of use. Users who engaged with GenAI for more than 2 hours per day reported higher AI literacy (mean difference 0.45-0.59, *P*=.001-.003) than other GenAI users, and they also showed higher life satisfaction (mean difference 1.60, *P*=.02) than nonusers.

### SEM

The original cascading model showed a mediocre fit (CFI=0.855, TLI=0.782, RMSEA=0.060, SRMR=0.048). There was a large modification index for a direct effect from behavioral intention to use AI to AI literacy. The modified SEM with the added effect showed a good fit (CFI=0.974, TLI=0.960, RMSEA=0.026, SRMR=0.036) to the data. As Table 5 shows, women showed lower AI literacy (β =–0.06, *P*=.005) than men. AI-related constructs showed negative associations with age (β=–0.22 to –0.40, *P*<.001) and chronic condition (β=–0.12 to –0.13, *P*<.001) and positive associations with education level (β=0.14-0.24, *P*<.001). Household income was positively associated with AI-related constructs and life satisfaction (β=0.07-0.23, *P*<.001). The eight covariates explained 5.4% to 44.2% of the total variance of the study variables.

**Figure 3:**
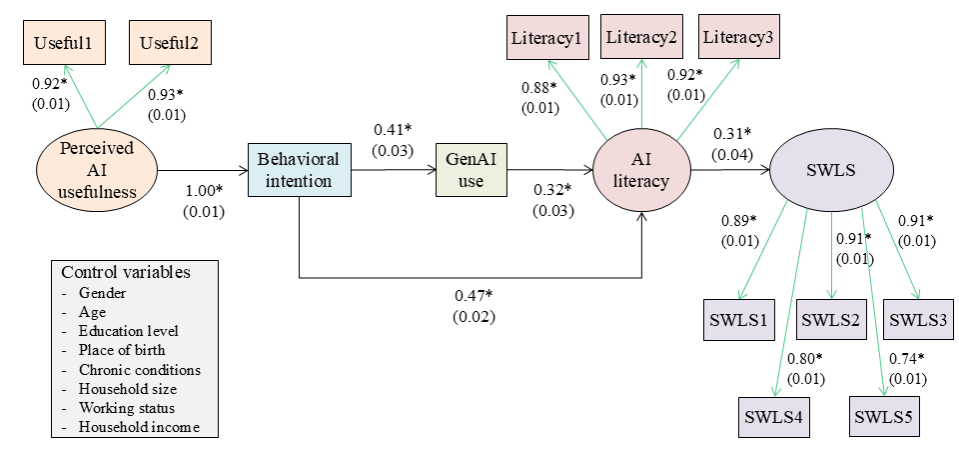
Standardized effects (SEs in brackets) in the structural equation modeling on perceived artificial intelligence usefulness, behavioral intention to use artificial intelligence, generative artificial intelligence use, artificial intelligence literacy, and life satisfaction in the sample. Factor loadings are shown in green arrows. *P<.01; effects of sociodemographic variables are shown in Table 5. AI: artificial intelligence; GenAI: generative artificial intelligence; SWLS: Satisfaction with Life Scale.

Figure 3 displays the SEM results for the main model, where the 3 latent factors showed substantial factor loadings (λ=0.74-0.93, *P*<.001). Controlling for the covariates, perceived AI usefulness had a very strong association (β=1.00, *P*<.001) with behavioral intention to use AI; behavioral intention to use AI was moderately associated (β=0.41, *P*<.001) with GenAI use; behavioral intention to use AI and GenAI use were moderately associated (β=0.32-0.47, *P*<.001) with AI literacy; and AI literacy was moderately associated (β=0.31, *P*<.001) with life satisfaction. The model explained an additional 64.8%, 10.5%, 29.5%, and 5.3% of the variance in behavioral intention to use AI, GenAI use, AI literacy, and life satisfaction, respectively.

**Table 5:** Association between sociodemographic variables and perceived AI^a^ usefulness, behavioral intention to use AI, GenAI^b^ use, AI literacy, and life satisfaction in the structural equation modeling.

Sociodemographic predictors	Perceived AI usefulness, β (SE)	Behavioral intention to use AI, β (SE)	GenAI use, β (SE)	AI literacy, β (SE)	Life satisfaction, β (SE)
Women	0.01 (0.02)	0.01 (0.02)	–0.06 (0.03)	–0.06 (0.02)^c^	–0.01 (0.03)
Age	–0.27 (0.04)^c^	–0.22 (0.04)^c^	–0.29 (0.04)^c^	–0.40 (0.03)^c^	0.10 (0.05)
Education level	0.24 (0.03)^c^	0.24 (0.03)^c^	0.14 (0.04)^c^	0.19 (0.03)^c^	0.02 (0.04)
Born in Hong Kong	–0.05 (0.02)	–0.04 (0.02)	0.06 (0.03)	–0.01 (0.02)	0.01 (0.03)
Chronic condition	–0.13 (0.03)^c^	–0.13 (0.03)^c^	–0.06 (0.04)	–0.12 (0.03)^c^	–0.07 (0.04)
Household size	–0.07 (0.02)^c^	–0.07 (0.02)^c^	–0.05 (0.03)	–0.04 (0.02)	–0.01 (0.03)
Work status	0.03 (0.03)	0.05 (0.03)	0.04 (0.04)	0.03 (0.03)	–0.03 (0.03)
Household income	0.16 (0.03)^c^	0.16 (0.03)^c^	0.13 (0.03)^c^	0.07 (0.02)^c^	0.23 (0.03)^c^
Total *R*^2^ (%)	37.6	34.3	29.0	44.2	5.4

^a^AI: artificial intelligence.

^b^GenAI: generative artificial intelligence.

^c^*P*<.01.

### Indirect Effects and Multigroup SEM Across Sociodemographic Subgroups

Multigroup SEM across gender, age groups, work status, and chronic condition provided adequate fit to the data (CFI=0.963-0.974, TLI=0.949-0.964, and RMSEA=0.025-0.030). All path coefficients showed no significant differences across work status and chronic condition. Across gender, the path of behavioral intention to use AI → AI literacy was significantly stronger (*P*=.02) in men (β=0.51) than in women (β=0.40); and the path of AI literacy → life satisfaction was significantly stronger (*P*=.02) in men (β=0.40) than in women (β=0.21). Across age groups, the path of GenAI use → AI literacy in middle-aged adults (β=0.47) was significantly stronger (*P*<.001) than in young adults (β=0.24) and significantly stronger (*P*=.01) than in older adults (β=0.33). The behavioral intention to use AI → AI literacy path was significantly stronger (*P*=.001) in young adults (β=0.60) than in middle-aged adults (β=0.41).

Table 6 lists the indirect effects of perceived AI usefulness on life satisfaction via behavioral intention to use AI, GenAI use, and AI literacy across sociodemographic subgroups. In the overall sample, perceived AI usefulness showed a significant positive total indirect effect (αβ=0.186, 95% CI 0.134-0.242) on life satisfaction. The indirect effects were consistent across work status and chronic conditions, with highly overlapping 95% CIs. Across gender, the indirect effects were substantially stronger in men (αβ=0.260, 95% CI 0.177-0.348) than in women (αβ=0.112, 95% CI 0.055-0.180). Across age groups, the indirect effects were substantially stronger in older adults (αβ=0.227, 95% CI 0.113-0.367) than in young adults (αβ=0.124, 95% CI 0.038-0.214).

**Table 6:** Standardized indirect effects of perceived AI^a^ usefulness on life satisfaction via behavioral intention to use AI, GenAI^b^ use, and AI literacy in the whole sample and across subgroups.

Subgroup variable		Perceived AI usefulness → Behavioral intention to use AI → GenAI use → AI literacy → SWLS^c^, αβ (95% CI)	Perceived AI usefulness → Behavioral intention to use AI → AI literacy → SWLS, αβ (95% CI)	Total indirect effect, αβ (95% CI)
Whole sample		0.040 (0.029-0.055)	0.146 (0.103-0.193)	0.186 (0.134-0.242)
Work status				
	Not working	0.032 (0.015-0.058)	0.141 (0.066-0.225)	0.172 (0.084-0.268)
	Working	0.040 (0.027-0.057)	0.130 (0.086-0.180)	0.170 (0.116-0.231)
Chronic conditions				
	No	0.036 (0.024-0.051)	0.131 (0.088-0.179)	0.168 (0.115-0.224)
	Yes	0.036 (0.017-0.086)	0.134 (0.049-0.236)	0.170 (0.075-0.289)
Gender				
	Men	0.059 (0.037-0.087)	0.201 (0.134-0.273)	0.260 (0.177-0.348)
	Women	0.026 (0.013-0.042)	0.087 (0.041-0.144)	0.112 (0.055-0.180)
Age groups				
	Young adults	0.017 (0.005-0.034)	0.107 (0.031-0.186)	0.124 (0.038-0.214)
	Middle-aged adults	0.056 (0.038-0.076)	0.118 (0.077-0.166)	0.173 (0.120-0.234)
	Older adults	0.036 (0.015-0.076)	0.191 (0.089-0.317)	0.227 (0.113-0.367)

^a^AI: artificial intelligence.

^b^GenAI: generative artificial intelligence.

^c^SWLS: Satisfaction with Life Scale.

## Discussion

### Principal Results

This study examined the age differences in perceived AI usefulness, behavioral intention to use AI, GenAI use, and AI literacy and their relations to life satisfaction in a population-based sample of Hong Kong adults in 2024. This study synthesized the simplified TAM and self-determination theory into the GenAI context and elucidated the interrelationships between the study variables. About Hypothesis 1, although women reported lower levels across all AI-related constructs, the difference was significant only in AI literacy but not in perceived AI usefulness, behavioral intention to use AI, and GenAI use. Consistent with Hypothesis 2, there was a substantial decline in GenAI use with age, which showed negative associations with perceived AI usefulness, behavioral intention to use AI, and AI literacy. The MANCOVA results found significantly higher levels of perceived AI usefulness and behavioral intention to use AI for the GenAI users over the nonusers, supporting Hypothesis 3. AI literacy and life satisfaction showed linear increasing trends across GenAI use levels in MANCOVA. These results provide empirical evidence for Hypothesis 4. Finally, an indirect association was found between GenAI use and life satisfaction via AI literacy, lending support to Hypothesis 5.

### Comparison With Prior Work

In the present sample, 38.5% (693/1800) of the respondents used GenAI in 2024, which was comparable to a 39% usage rate among US respondents aged 18-64 years at the same time [38]. In contrast to the finding of a meta-analytic review [39], men in the present sample reported higher AI literacy than women. The gender discrepancy could be attributed to reasons such as higher education level and working proportion. Besides, men tend to overestimate their AI knowledge and skills while women tend to underestimate themselves [40]. This study found substantial age differences in GenAI use, with the usage rate dropping from more than half in young adults to less than one-sixth in older adults. The negative association of age with GenAI use and AI-related constructs remained significant after controlling for known determinants (education level and socioeconomic status) [41]. The robust associations likely reflect generational differences in technology receptiveness and familiarity and could be attributed to higher openness personality trait in younger generations [42] and greater technology anxiety in older adults [17].

In line with similar studies [5,6,43], the SEM found a strong association between subjective perceptions of AI and behavioral intention to use AI, and the latter served as a pathway through which perceived AI usefulness was indirectly associated with GenAI use. Through GenAI use, users could gradually familiarize themselves with GenAI tools, acquire skills in human-AI communication and prompting, and develop critical thinking abilities to recognize stereotypes potentially induced by GenAI [44]. Self-determination theory suggests that learning is more effective when driven by autonomous motivation rather than external pressure, leading to greater engagement and better outcomes in GenAI use [10]. People with higher AI literacy would compare outputs across multiple GenAI platforms, adopt a critical disposition towards the outputs’ reliability, and verify information through lateral reading [45].

This study provides a nuanced understanding of GenAI use and life satisfaction. Our study found a positive but small relationship between GenAI use and life satisfaction, which is consistent with previous findings [15,16]. The direct effect between GenAI use and life satisfaction became nonsignificant after accounting for AI literacy in the SEM. In the SEM, a path was added from behavioral intention to use AI to AI literacy because of high modification index. This added path aligns with self-determination theory in which autonomous and self-directed learning results in higher AI competence and better digital well-being [46]. Besides, the bridging role of AI literacy as an indirect statistical pathway was robust across sociodemographic subgroups. These results underscore that GenAI use is associated with life satisfaction predominantly via its relationship with AI literacy. Existing studies have linked general self-efficacy with higher life satisfaction [47,48]. AI literacy has shown a mediating role in critical thinking and future work readiness in education settings [49,50] via factors such as greater adaptability, problem-solving skills, and digital confidence [51,52].

Subgroup analysis revealed preliminary findings on potential heterogeneity in the indirect effects across gender and age groups. The gender discrepancy in the indirect effects of AI literacy is in line with the results of a meta-analysis [26]. Men are typically overrepresented in Science, Technology, Engineering, and Mathematics–related fields and have greater exposure to on-the-job training in AI literacy as reflected in their higher levels of AI literacy than women. Women are more likely to face systemic barriers, such as algorithmic bias that tends to marginalize them, resulting in a digital divide and a smaller mediating role of AI literacy for them [25]. Furthermore, AI literacy mainly taps into the psychological needs of autonomy and competence while women value relatedness more in the evaluation of life satisfaction [53].

Despite lower levels of GenAI use and AI literacy among older adults, our study found a stronger bridging role for AI literacy among this age subgroup than among young adults. This discrepancy may appear counterintuitive since young adults are considered digital natives. The higher baseline digital literacy in young adults could denote a ceiling effect where AI literacy plays a lesser role, and AI literacy could provide greater marginal benefits to older adults given their lower digital fluency at baseline. Older adults are more risk-averse about data privacy and security issues than young adults, and AI literacy could play a greater role in helping them to trust and understand the rules on GenAI use. However, the very small number of older adults who used GenAI for more than 2 hours per day could make the subgroup results less stable and more vulnerable to sampling error. Given the exploratory nature of the subgroup analyses, future research should clarify the age differences in the bridging role of AI literacy in the relationship between GenAI use and life satisfaction in other samples.

### Future Work

Findings have some implications for digital-literacy policy and support targeted programs to mitigate digital divides among sociodemographic subgroups. Policymakers should strive for digital inclusion and active literacy cultivation in the digital age and ensure equitable access to GenAI among those with lower socioeconomic status [54]. Hands-on assistance from family and peers should be encouraged to build AI literacy, which was found to mitigate the relationship between perceived anthropomorphism and fear of AI [55]. Further enhancements of the usability of GenAI, in terms of accuracy, transparency, and reliability, could build up related literacy and promote its implementation into health care information seeking and self-care management among the general public [56]. As women tend to underestimate their digital literacy while their objective digital capabilities could be as good as men’s [24,25], their self-efficacy and sense of control in using AI could be promoted. Education on using GenAI for older adults could focus more on digital content creation, safety, and problem-solving ability, and GenAI tools can be developed with less emotional distance and age-friendliness to build their trust [57].

### Limitations

This study had a few limitations. First, the current cross-sectional design did not allow causal inference about the effects of GenAI use on life satisfaction. Respondents who felt more satisfied with life could be more likely to use GenAI, and higher AI literacy could predict more frequent GenAI use. Longitudinal studies are needed to confirm the mediating role of AI literacy and clarify the reciprocal effects of AI literacy and life satisfaction on GenAI use. Second, this study used short forms of validated scales (AIAS-4 and AILS) to measure perceived AI usefulness, behavioral intention to use AI, and AI literacy. These adapted scales showed strong factor loadings and measurement invariance across demographic subgroups in the present sample, which lent support to their construct validity. However, the current 3-item brief version did not include any items from the Ethics subscale of AILS. This raises potential concerns over the content validity of the adapted measure. Further research is needed to elucidate the ethical domain of AI literacy in the relationship with GenAI use and life satisfaction. Third, this study only measured the respondents’ GenAI use using a single self-reported item on their daily time. The single-item measure for GenAI use could not account for potential measurement errors and may introduce biases in the results. Besides, we did not assess the type of GenAI tool, purpose, and accuracy of use [11]. Further research is required to evaluate the antecedents and contextual determinants of ethical use of GenAI.

Fourth, the nonresponses in this study could be due to lower technological engagement and enthusiasm toward GenAI. This would induce a positive self-selection bias that would overestimate GenAI use and AI literacy in the sample. Unequal selection probabilities at geographic or household levels were not accounted for, leading to overrepresentation of the individuals living alone or in smaller districts. The use of self-report measures denotes common method bias. Fifth, this study focused on positive attributes of AI (usefulness and literacy) and did not assess fear of AI as a barrier to GenAI adoption. Technology anxiety has shown significant relationships with acceptance, literacy, and attitudes toward AI [58]. Compulsive use of ChatGPT has shown a negative indirect effect on life satisfaction via technostress [59]. Further studies should evaluate the role of technology anxiety and technostress in GenAI adoption for health-enhancing purposes. Six, this study did not include other psychological factors of life satisfaction, such as locus of control, intolerance of uncertainty, hope, and resilience. The omitted variables could lead to confounding bias in the present results. Future studies should consider these factors as alternative pathways that help explain the relationship between GenAI use and life satisfaction.

### Conclusions

This study found relatively low GenAI use among Hong Kong adults in 2024, with a notable decreasing trend in GenAI use and AI literacy across age groups, especially among older adults. The findings showed that AI literacy was statistically positioned in the positive indirect pathway between GenAI use and life satisfaction and longitudinal studies are needed to test the causal direction of the relationships. These findings have potential implications for informing digital health initiatives and policies to leverage GenAI tools.

## Abbreviations

AI: artificial intelligence
AIAS-4: 4-item Artificial Intelligence Attitude Scale
AILS: Artificial Intelligence Literacy Scale
BIC: Bayesian information criterion
CFA: confirmatory factor analysis
CFI: comparative fit index
GenAI: generative artificial intelligence
MANOVA: multivariate analysis of variance
RMSEA: root-mean-square error of approximation
SEM: structural equation modeling
SRMR: standardized root-mean-square residual
STROBE: Strengthening the Reporting of Observational Studies in Epidemiology
SWLS: Satisfaction with Life Scale
TAM: Technology Acceptance Model
TLI: Tucker-Lewis index

## Data Availability

The datasets generated or analyzed during this study are available from the corresponding author on reasonable request.
